# The first genotype II African swine fever virus isolated in Africa provides insight into the current Eurasian pandemic

**DOI:** 10.1038/s41598-021-92593-2

**Published:** 2021-06-22

**Authors:** Emma P. Njau, Jean-Baka Domelevo Entfellner, Eunice M. Machuka, Edwina N. Bochere, Sarah Cleaveland, Gabriel M. Shirima, Lughano J. Kusiluka, Chris Upton, Richard P. Bishop, Roger Pelle, Edward A. Okoth

**Affiliations:** 1 Biosciences Eastern and Central Africa-International Livestock Research Institute Hub, Nairobi, Kenya; 2 Nelson Mandela African Institution of Science and Technology, Arusha, Tanzania; 3 Institute of Biodiversity, Animal Health and Comparative Medicine, College of Medical, Veterinary and Life Sciences, University of Glasgow, Glasgow, UK; 4 Sokoine University of Agriculture, P. O. Box 3019, Morogoro, Tanzania; 5 Mzumbe University, Morogoro, Tanzania; 6 Biochemistry and Microbiology, University of Victoria, Victoria, BC V8W 3P6 Canada

**Keywords:** Bioinformatics, Genomic analysis, Sequencing, Computational biology and bioinformatics, Molecular biology, Pathogens, Virology

## Abstract

African swine fever (ASF) caused by the African swine fever virus (ASFV) is ranked by OIE as the most important source of mortality in domestic pigs globally and is indigenous to African wild suids and soft ticks. Despite two ASFV genotypes causing economically devastating epidemics outside the continent since 1961, there have been no genome-level analyses of virus evolution in Africa. The virus was recently transported from south-eastern Africa to Georgia in 2007 and has subsequently spread to Russia, eastern Europe, China, and south-east Asia with devastating socioeconomic consequences. To date, two of the 24 currently described ASFV genotypes defined by sequencing of the p72 gene, namely genotype I and II, have been reported outside Africa, with genotype II being responsible for the ongoing pig pandemic. Multiple complete genotype II genome sequences have been reported from European, Russian and Chinese virus isolates but no complete genome sequences have yet been reported from Africa. We report herein the complete genome of a Tanzanian genotype II isolate, Tanzania/Rukwa/2017/1, collected in 2017 and determined using an Illumina short read strategy. The Tanzania/Rukwa/2017/1 sequence is 183,186 bp in length (in a single contig) and contains 188 open reading frames. Considering only un-gapped sites in the pairwise alignments, the new sequence has 99.961% identity with the updated Georgia 2007/1 reference isolate (FR682468.2), 99.960% identity with Polish isolate Pol16_29413_o23 (MG939586) and 99.957% identity with Chinese isolate ASFV-wbBS01 (MK645909.1). This represents 73 single nucleotide polymorphisms (SNPs) relative to the Polish isolate and 78 SNPs with the Chinese genome. Phylogenetic analysis indicated that Tanzania/Rukwa/2017/1 clusters most closely with Georgia 2007/1. The majority of the differences between Tanzania/Rukwa/2017/1 and Georgia 2007/1 genotype II genomes are insertions/deletions (indels) as is typical for ASFV. The indels included differences in the length and copy number of the terminal multicopy gene families, MGF 360 and 110. The Rukwa2017/1 sequence is the first complete genotype II genome from a precisely mapped locality in Africa, since the exact origin of Georgia2007/1 is unknown. It therefore provides baseline information for future analyses of the diversity and phylogeography of this globally important genetic sub-group of ASF viruses.

## Introduction

**Figure 1:**
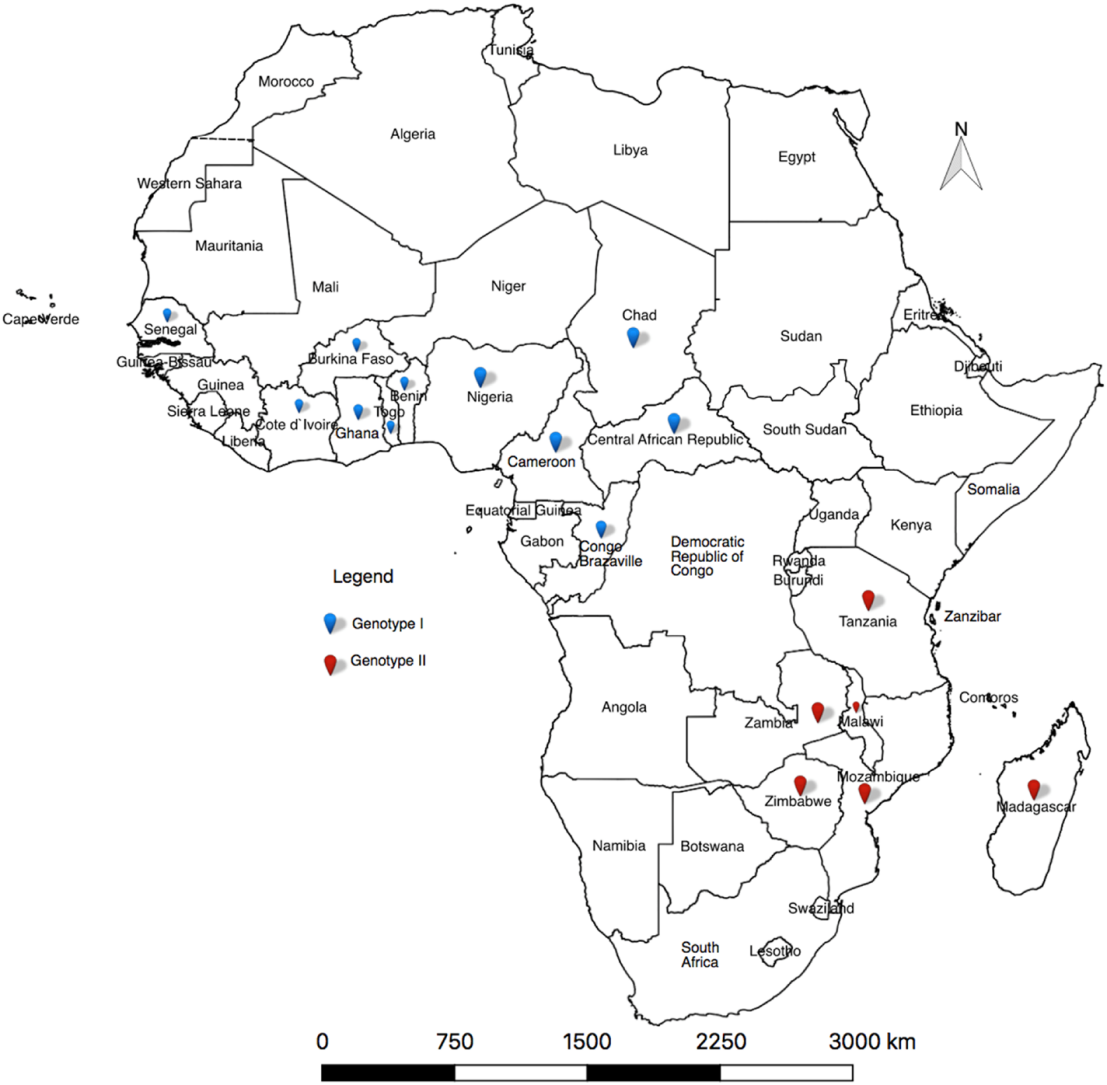
A map showing the geographical distribution of the two genotypes that have escaped outside Africa into other continents. While genotype I has dominated the western, genotype II is more prevalent in the eastern part of Africa. This map was constructed using QGIS software version 2.18.14 freely downloaded from https://qgis.org/en/site/.

According to the World Organization for Animal Health (OIE), African swine fever virus (ASF) is the most important disease-causing pathogen affecting the domestic swine population globally^1^. The high mortality induced in naïve pig populations is devastating to pig farmers, especially given that there is no chemotherapy or vaccine currently available for disease control. Prevention of the disease relies on strict biosecurity measures which are frequently not effectively applied, particularly in the endemic areas. As a result, the virus and associated disease have spread into many new areas that have not previously been reported (reviewed by^2–4^). Historically, the disease was first described in East Africa in the 1920s^5^. The first occurrence outside Africa, of a virus classified in p72 genotype I, was reported in 1958 and again in 1961 from Lisbon Portugal, with subsequent spread to other regions of Europe and Latin America (reviewed by^6,7^). In 2007, an ASF virus (ASFV) within p72 genotype II^8^ was again exported through a human agency outside sub-Saharan Africa into Georgia. However, the precise geographical origin has not been identified, since genotype II is present in Mozambique, Madagascar, Malawi, Zambia, Zimbabwe and Southern Tanzania^8–16^ as represented in Fig. 1.

Using a risk assessment methodology, shipment of a pork product originating from southeastern Africa, or Madagascar was highlighted as a likely source of viruses that could be disseminated into other continents^17^.

Between mid-2007 to date, ASF genotype II viruses, derived from the Georgia introduction, have been reported throughout the Caucasus, Russia, the Baltic republics, Czech Republic, Romania, Hungary, Bulgaria, Poland, Belgium, and most recently China^18^ and South-east Asia. Despite significant efforts by FAO and others to control its spread, numbers of ASFV infected animals are increasing rapidly in domestic pigs and wild boar populations, with movements of the latter facilitating the rapid geographical spread of the virus (reviewed in^2^).

Constituting the sole member of the genus *Asfivirus* within the family *Asfiviridae*, ASFV is the only known DNA virus causing hemorrhagic fever transmitted by an arthropod, namely Argasid soft ticks in the genus *Ornithodoros*. The genome of the virus is composed of a linear double-stranded DNA molecule between 175 and 195 kbp in length and containing up to 190 open reading frames depending on the virus isolate^19^. Multiple approaches have been used to characterize the virus^20^, with genotyping of the virus by full length or partial sequencing of the genes encoding the p72, p54, p30 proteins and the Central variable region now widely applied.

Twenty-four genotypes of ASFV have been identified by sequencing the p72/B646L gene of the virus^21,22^. Most of these genotypes can be associated with particular geographical regions, especially in sub-Saharan Africa, where the virus almost certainly originated, and is endemic in wild suids^23^. Among the 24 published genotypes, genotype II is currently of the highest global importance, since it is the cause of an ongoing pig pandemic encompassing a region from Eastern Europe to China and Southeast Asia. The economic losses involved are enormous, amounting to as much as 25% of global pig production. The socioeconomic impact is particularly severe in China, which produces 450 million of the world’s pigs, many of which are in small or medium scale, backyard or village systems^18^.

Multiple complete genome sequences of ASFV have been determined with a strong bias towards p72 genotypes 1 and II. However, with a few exceptions, for example, Benin and South African isolates in p72 genotype 1^24,25^, most of the complete genomes are derived from isolates sampled outside Africa in novel environments into which ASFV has recently been introduced by human agency. The newly infected agroecosystems in Eurasia lack both the ancestral tick vector (*Ornithidorus moubata/porcinus*) and also warthogs (*Phacocherus africanus*) and bush pigs (*Potamochoerus larvatus*), the mammalian hosts with which the viruses have co-evolved over millions of years in the African continent. The currently available complete genomes derived from ASFV genotypes I and II are therefore of limited relevance for reconstructing the evolutionary history of ASF viruses. The complete genome sequence of an African ASFV genotype II virus, has not yet been reported, despite the devastating impact of this genotype on domestic pig production across Eurasia^18,26–32^. To date, the analyses of genotype II within Africa have been confined to genotyping of the viruses at selected polymorphic loci, particularly the 3ʹ end of the gene encoding the p72 surface protein (locus B646L) where most mutations sampled appear synonymous at the protein level^33^. The p72 gene analysis is typically combined with sequencing the region encoding the tetrapeptide central variable region (CVL) within locus p602L, which provides high-resolution data for differentiating lineages that have emerged on short timescales. Such data, as seen in Supplementary Figure S1, while useful for underpinning control by monitoring outbreaks and tracking virus movement, is inadequate for an in-depth understanding of the evolutionary history dynamics and functional changes that have occurred in this ASFV lineage. In the current study, we present the first fully annotated genome sequence of a geo-referenced genotype II ASF virus from Africa, isolated post mortem from a clinically ill domestic pig in the Rukwa region of southwest Tanzania, together with a comparative analysis with other ASFV genomes derived from Georgia/2007/1. This annotated ASFV genome will provide an initial reference point for subsequent research on genotype II evolution and dynamics in Africa.

## Results

### Virus isolation and initial characterization

We generated the whole genome sequence of a p72 genotype II ASFV that was isolated from a clinically ill domestic pig in the Rukwa region of the southern highlands of Tanzania following infection of PBMCs using a virus suspension prepared from spleen tissue. The location within Tanzania of the district from which the virus was isolated and its geographical separation from the presumptive origin of Georgia 2007/1 in Southeastern Africa is shown in Fig. 6. The virus was demonstrated to be hemadsorbing, since after infection of porcine PBMC with virus-containing lysate at an MOI of 10:1, formation of rosette structures was observed following addition of homologous red blood cells to an infected culture. Additional confirmation of the presence of ASFV in the culture was obtained by conventional PCR using p72 primers that generated an amplicon of 257 bp the expected size for ASFV. The positive cultures were passaged only once to minimize the risk of mutation of the virus. Sequencing of the C-terminal end of the p72, p54, p30 gene and the CVR indicated that these viruses were within ASFV genotype II as seen in the Supplementary Figures S1–S4. Three field isolates were obtained using this procedure, although only one sample, designated Tanzania/Rukwa/2017/1, resulted in a complete genome sequence following assembly of the raw data.

### Comparative analysis of the Tanzania/Rukwa/2017/1 with other ASFV genotype II complete genomes

**Figure 2:**
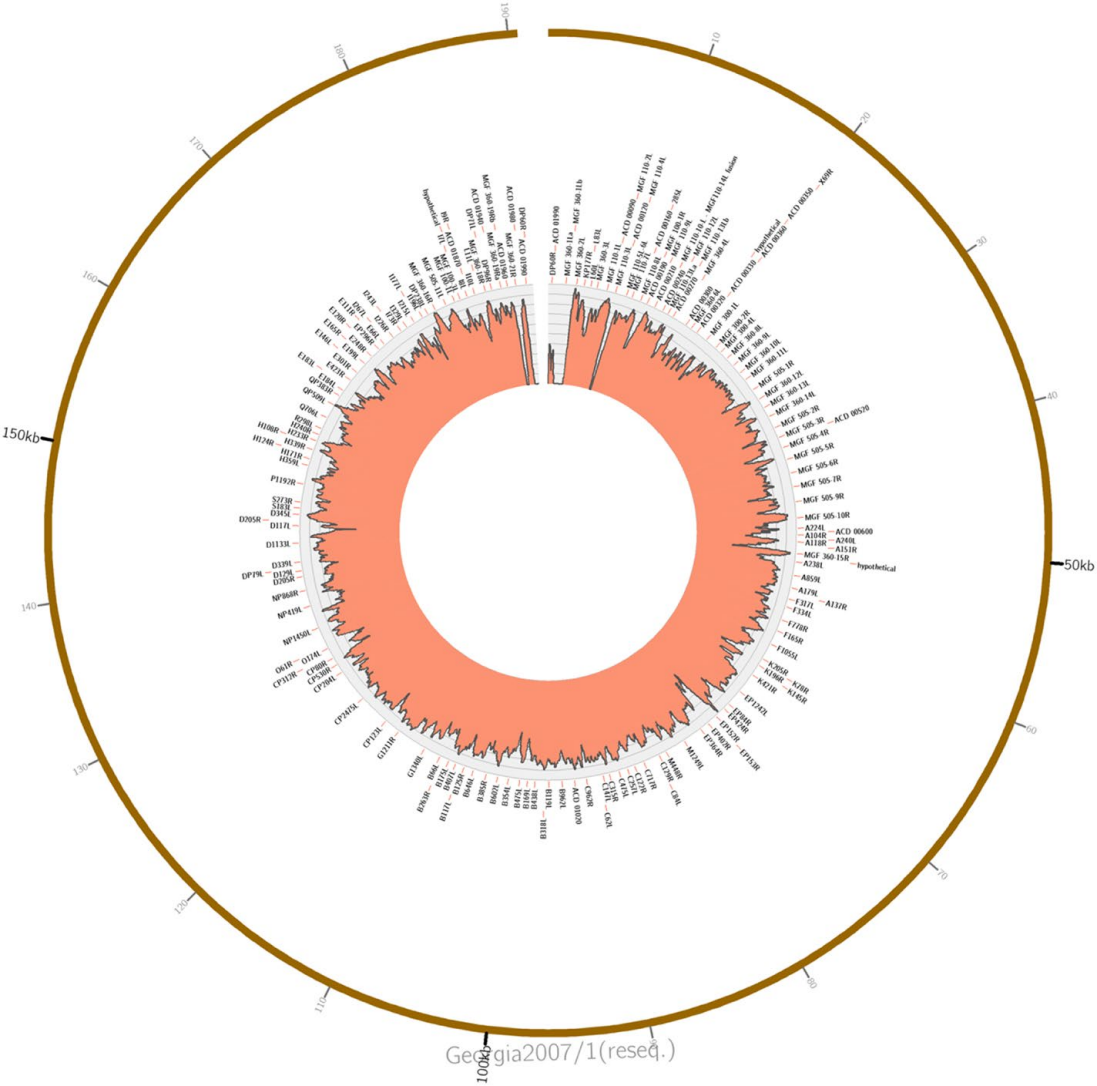
Circos plot representing the mapping of reads from the Tanzania/Rukwa/2017/1 genome to the recently corrected Georgia 2007/1 (FR682468.2) strain. The histogram shows log read depth values. The inner wheel gives approximate locations for the open reading frames.

For comparative genomics analysis, reference genotype II whole genome sequences published in previous studies were retrieved from the European Nucleotide Archive. While all publicly available genotype II genomes were closely related to the Tanzania/Rukwa/2017/1 sequence, we concentrated on three that were representative of different geographical regions spanning the extent of the current pandemic of ASFV across Eurasia. These were the original genotype II isolate sampled outside of Africa, considered to represent the index case of the current pandemic affecting Asia and Europe (Georgia 2007/1, formerly sequenced and published as FR682468.1, and recently re-sequenced to produce the published sequence FR682468.2—the version used herein), the Polish virus Pol16_29413_o23 (MG939586.1, isolated in 2016/2017) and a Chinese isolate ASFV-wbBS01 sampled in 2018 (MK645909.1). Initial comparison of the new Tanzanian viral genome determined in this study was performed by mapping with the minimap2 program to the Georgia/2007/1 isolate (FR682468.2). A Circos plot representing the mapping of reads to the recently corrected Georgia 2007/1 (FR682468.2) genome sequence was drawn and is presented in Fig. 2. This analysis reveals good coverage of the Tanzania/Rukwa/2017/1 relative to the Georgia 2007/1 reference. This mapping analysis was similar to one generated using the Bowtie2 program for comparison with the Chinese ASFV-wbBS01:MK645909.1.

### Comparison of Tanzania/Rukwa/2017/1 genome sequence with the re-annotated Georgia 2007/1 ASFV genotype II reference genome

**Figure 3:**
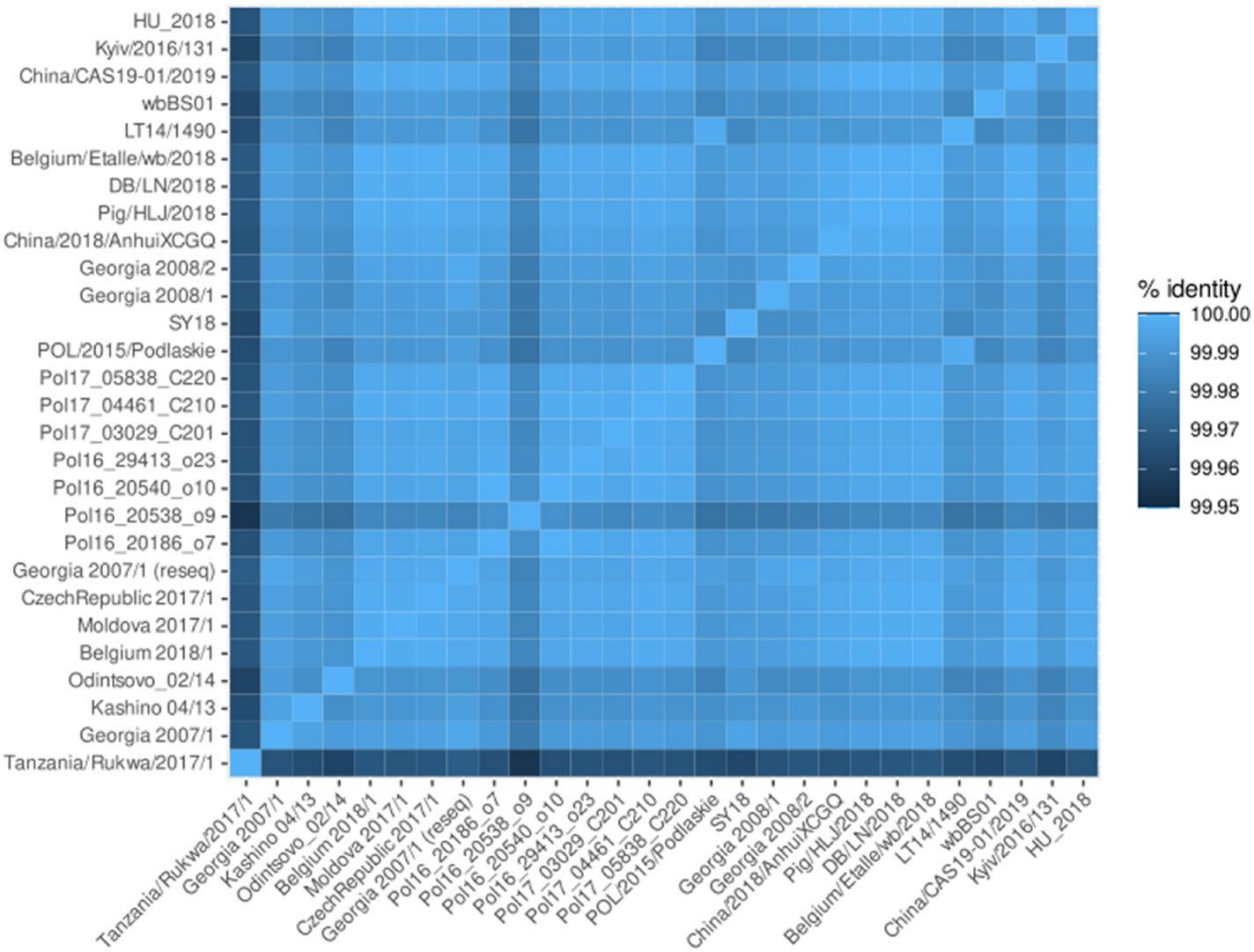
Genome-wide pairwise sequence identities between genotype II viruses. The highly dissimilar isolate Estonia 2014 (accession number LS478113.1) is not included here. Gap-containing sites were ignored in sequence alignments in a pairwise manner.

The overall sequence similarity between the two genomes based on an un-gapped alignment was 99.671% and the relationship between Tanzania/Rukwa/2017/1 and other ASFV genotype II complete genomes are shown as a matrix of pairwise identity values in Fig. 3.

Since indels represent the major sources of variation between ASFV genomes, we focus primarily on this aspect in detail here. Major differences included the apparent absence of the single-copy locus ASFV_G_ACD_00120 from the Tanzania/Rukwa/2017/1 genome and a series of differences in 5ʹ and 3ʹ multicopy gene families (MGFs). Regarding the MGFs, specific differences include: (1) at the 5ʹ end of the Tanzania/Rukwa/2017/1 virus, the MGF360-1L gene is absent; (2) there is almost complete loss of MGF360_21R near to the 3ʹend of the Tanzania/Rukwa/2017/1 genome; (3) the copy of MGF110-7L in Tanzania/Rukwa/2017/1 is truncated (and therefore presumably non-functional) relative to Georgia 2007/1 (FR682468.2) and encodes only 39 amino acids, due to the insertion of a G resulting in a frameshift and introduction of a stop codon; (4) a deletion of one nucleotide in MGF360-14L results in a major truncation of this gene compared to Georgia 2007/1. (5) Two SNPs affect MGF505-4R in Tanzania/Rukwa/2017/1, the second one introducing a premature stop codon, so the length of this ORF is 369 aa versus 506 aa in Georgia/2007/1.

One indel not associated with the MGFs is an insertion of 16 bases that yields a tandem repeat (aaaaaaataaacaaca) located just 5ʹ of the ATG codon of locus KP177R as presented in the Supplementary Figure S5, which encodes the K177R structural protein. Analysis of the numbers of reads confirmed the presence of this mutation which differentiated Rukwa/2017/1 from all currently available genotype II genome sequences. In addition to the multiple changes in the terminal multicopy gene families, the single-copy gene ASFV_Ch_ACD_00120 appears to be absent from Tanzania/Rukwa/2017/1 but is present in Georgia 2007/1 and its derivatives.

It is noteworthy that the final inverted terminal repeat of Georgia 2007/1 (roughly from position 189,200 of the original reference genome FR682468.1) has abnormally high coverage of more than 1000×. One possible explanation is that sequential repeats have collapsed during assembly of both the ‘original’ reference Georgia 2007/1 genome (FR682468.1) and the re-sequenced Georgia 2007/1 (FR682468.2), but to a lesser degree. This problem of lack of resolution of the copy number in the terminal repeat region of ASFV has been noted previously^34^ and will be efficiently addressed only by long-read sequencing including the full terminal hairpin loops.

### Comparative analysis of Tanzania/Rukwa/2017/1 relative to Polish and Chinese genotype II genome sequences

Searches using BLAST indicated that genome sequence of Tanzania/Rukwa/2017/1 was very closely related to both the Chinese and Polish viruses. Pol16_29413_o23 (GenBank accession MG939586.1^31^) exhibited 99.960% similarity to Tanzania/Rukwa/2017/1 and the Chinese virus ASFV-wbBS01 (MK645909.1^28^) exhibited 99.957% similarity. The Tanzania/Rukwa/2017/1 sequence is noticeably shorter than the Polish sequence (183,186 bp as compared to 189,393 bp, i.e. shorter by 6207 bp). This is because the 5ʹ (3090 bp) and 3ʹ (2356 bp) overhang ends that are present in the Polish genome, could not be sequenced using our protocol. A number of indels also differentiated the Tanzanian and Polish virus genomes, including a long deletion of 688 bp close to the 5ʹ end of the Tanzania/Rukwa/2017/1 virus. Additionally, nucleotides 7423 to 8110 of the Polish virus (sites 7441 to 8128 in the alignment) are apparently absent in Tanzania/Rukwa/2017/1. There is also an insertion of 16 bp (taaacaacaaaaaaaa) at alignment positions 3250 to 3265 between nucleotides 3249 and 3250 of the Polish virus. Other deletions observed included 11 bp corresponding to nucleotides 25,319 to 25,329 of the Polish virus (sites 25,342 to 25,352), 66 bp corresponding to nucleotides 175,453 to 175,518 of the Polish virus (site 175,478 bp). The remaining shorter insertion and deletions range from one to six nucleotides in length and are located primarily in homopolymer runs throughout the assembly.

Additional differences are SNPs scattered across the genome; for instance at position 72,763 of the reference, we have a C/T SNP; G/A at position 70,664 and (A/G) at position 9711 of the reference. Other features include an insertion of one base (G) between positions 9660 and 9661 of the Polish genome Among the 189,420 nucleotide sites present in the pairwise alignment of Tanzania/Rukwa/2017/1 with Pol16_29413_o23, there are 183,159 complete sites (with no gaps and no undetermined nucleotides), among which there are only 73 variable sites. In total, we counted 126 SNPs from the mapping of our short reads to the Polish virus sequence MG939586.1.

The alignment of Tanzania/Rukwa/2017/1 with the Chinese ASFV-wbBS01 (accession: MK645909) comprises 189,427 sites, with 183,153 complete sites (without gaps or undetermined nucleotides), among which only 78 are variable. Unlike the Polish viral genome, the Chinese strain is annotated, with 185 predicted CDS and corresponding conceptual translations. At the 5ʹ end of the genome, reads mapping to the inverted tandem repeat region (nucleotides 1 to 422 of MK645909) were present. This was followed by a gap without reads in Tanzania/Rukwa/2017/1 corresponding to base pairs 400 to 2175 of MK645909. As already mentioned, MGF360-1L is missing in the Tanzanian isolate as compared to Georgia 2007/1 and viruses derived from the index isolate. However, the next representative of the 360-multigene family, encoded at the 5ʹ end of ASFV, MGF360-2L, is present downstream in Tanzania/Rukwa/2017/1. Two intergenic inserts of one base each were observed in Tanzania/Rukwa/2017/1 between MGF360-3L and MGF110-1L. There is also insertion of a T in Tanzania/Rukwa/2017/1 between positions 5818 and 5819 in MK645909 and insertion of a G in Tanzania/Rukwa/2017/1 between sites 6050 and 6051. There is a T/C SNP towards the beginning of MGF110-1L (position 6102 of the MK645909) corresponding to the second position with the TAG stop codon which becomes a tryptophan codon TGG, hence extending MGF110-1L from 196 amino acids in ASFV-wbBS01, to 269 a.a. in Tanzania/Rukwa/2017/1. The MGF110-1L open reading frame is also affected by two SNPs: C/T at position 6365 and A/T at position 6367 of the MK645909. These two SNPs are respectively in the third and first positions of a codon. The codon TGG (tryptophan) in wbBS01 is mutated to encode arginine (AGA) in Tanzania/Rukwa/2017/1. Additionally, MGF110-3L is truncated at the 5ʹ end. Within MGF110-4L, there are also SNPs, specifically T/C at position 8160 and C/A at position 8204. In the fused MGF 110 protein MGF110-5L-6L, a deletion of three nucleotides (ATG) across a codon boundary in Tanzania/Rukwa/2017/1, corresponding to positions 8670 to 8672 in wbBS01 was observed. In respect of the multicopy gene families located at the 3ʹ end of the genome, as already mentioned above, MGF360-21R is almost entirely deleted from the Tanzania/Rukwa/2017/1, but it is present in Georgia 2007/1 and the Polish and Chinese viruses derived from it over the last 10 years.

In EP152R, an essential protein that has been shown to interact with the host protein BAG6^35^ a non-synonymous SNP (T/C), is present at position 72,854 of MK645909, resulting in the substitution of Phe by Ser in Tanzania/Rukwa/2017/1. At the 3ʹ end of the virus, gene DP60R is altered by two single-nucleotide deletions and one SNP. These are the deletion of one A (from an A^10^ homopolymer in wbBS01) at position 189,148, followed by deletion of another A (from an A^9^ homopolymer in wbBS01) at position 189,172 and a SNP (A to G) at position 189,218. The comparative analysis of Belgium 2018/1 and Georgia 2007/1 also detected one such deletion/insertion of an A in the same homopolymer region of DP60R^34^.

### Phylogenetic analysis

**Figure 4:**
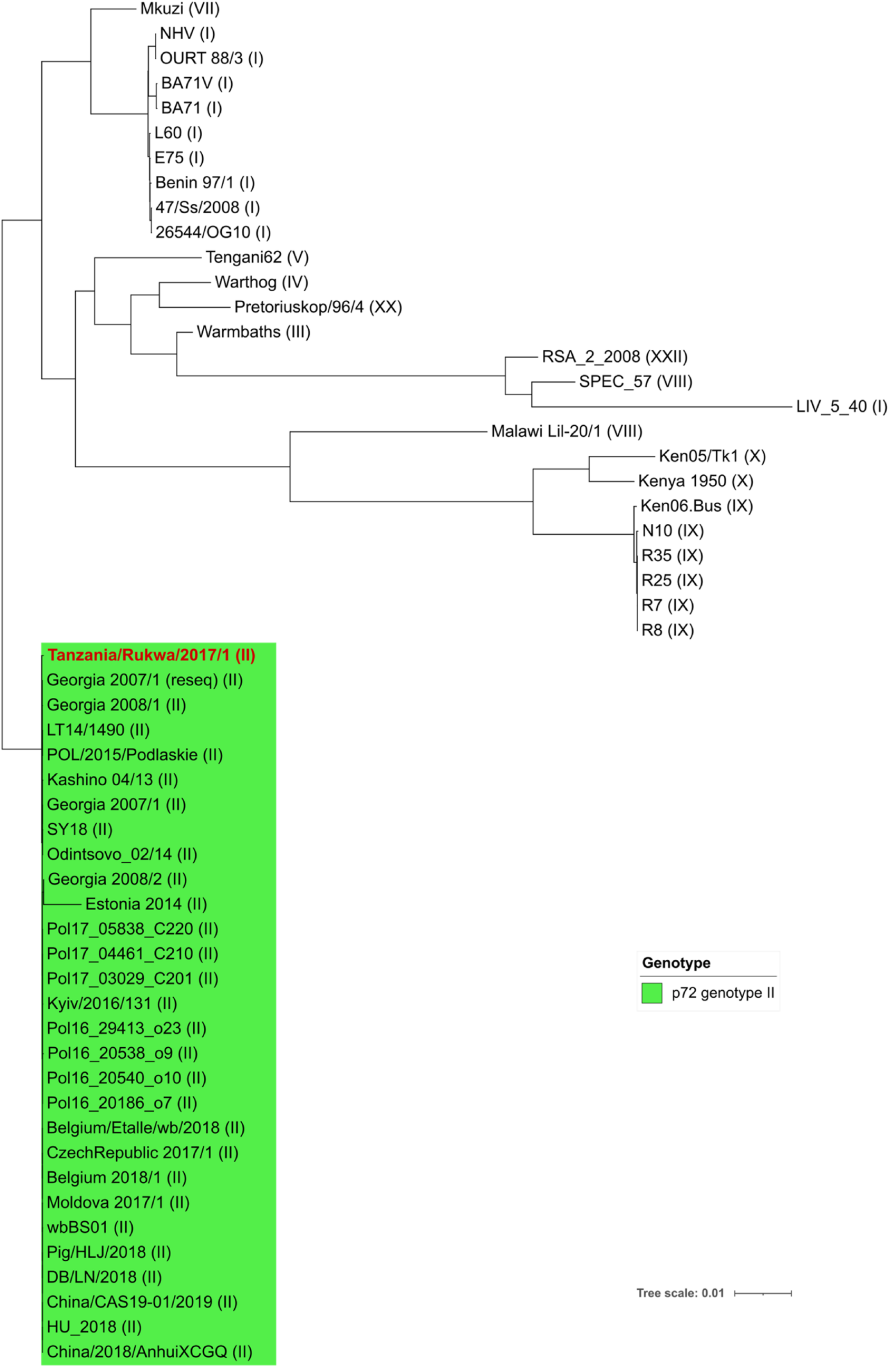
Maximum-likelihood phylogenetic tree showing the relationship between the sequence of Tanzania/Rukwa/2017/1 determined in this study (highlighted in red), and 54 additional full-length ASFV sequences retrieved from the public databases. The branch length unit is the number of expected substitutions per site.

A total of 54 additional full-length ASFV genome sequences obtained from GenBank was used to generate a maximum-likelihood tree (using FastTree with a GTR evolutionary model). The tree is depicted in Fig. 4.

The tree was inferred from full-genome sequences: gappy and hypervariable (saturated) sites were trimmed out using BMGE, and FastTree was used to generate a phylogenetic tree with a GTR evolutionary model. The p72 genotypes are indicated with roman numerals between parentheses.

This phylogeny, generated using whole genome sequence data, clearly places Tanzania/Rukwa/2017/1 as a member of the genotype II ASFV taxonomic unit, a monophyletic clade is highly conserved, except for Estonia 2014 (accession LS478113.1), which displays some divergence. The differences in the Estonia sequence may be attributable to several indels not shared with other ASFV genotype II viruses (see for instance^36^ for a partial analysis of this).

**Figure 5:**
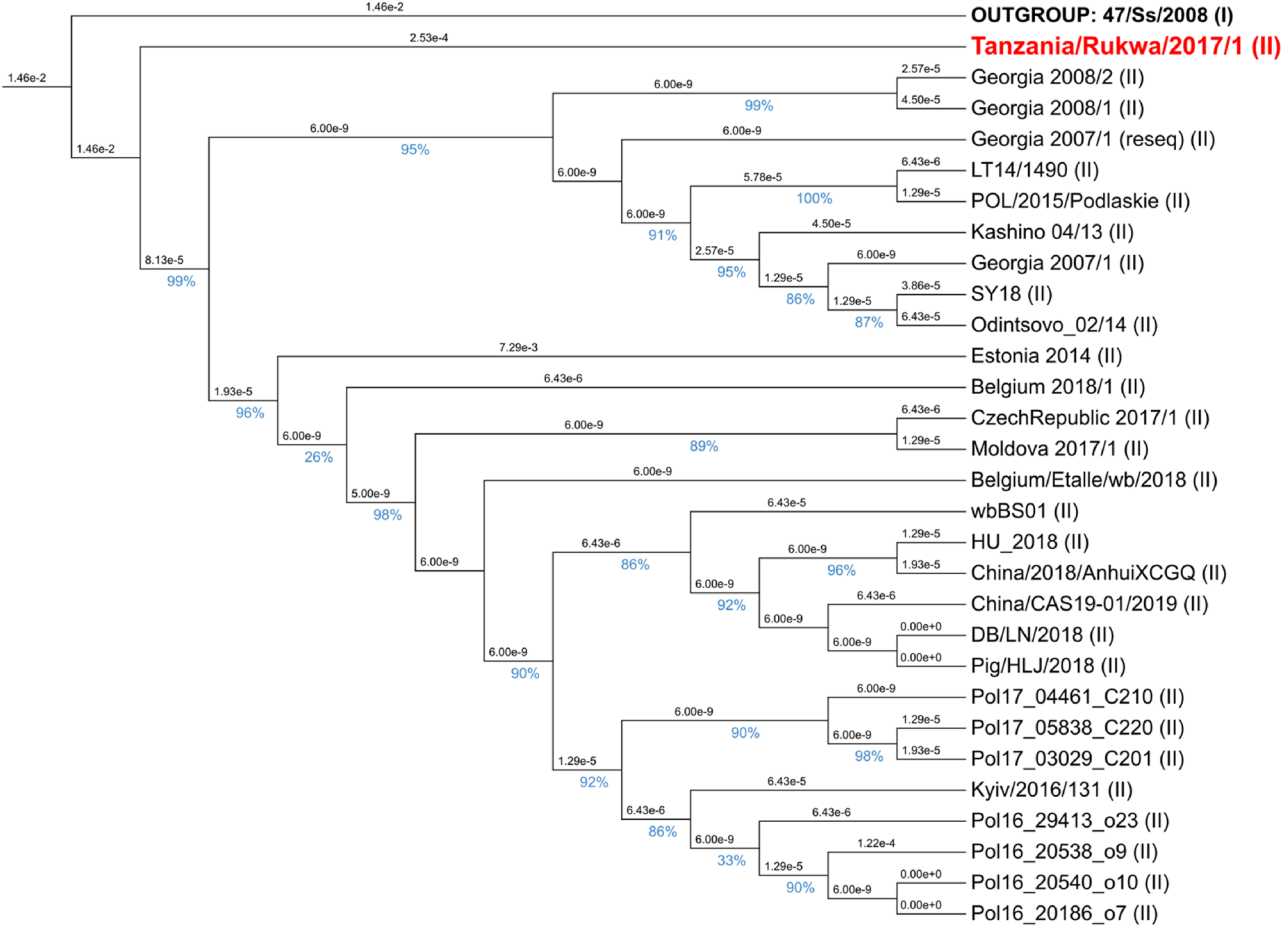
Maximum-likelihood phylogenetic tree of 29 complete genotype II genomes rooted with a genotype I outgroup sequence from Sardinia. The tree is represented as a cladogram instead of a phylogram for the sake of legibility. Branch lengths, expressed in number of expected substitutions per site, are displayed in scientific notation above the branches. Local Shimodaira-Hasegawa (SH-like) statistical branch supports calculated by FastTree are displayed as percentages in blue colour below the branches. We notice the high support (99%) attributed to the branch that makes Tanzania/Rukwa/2017/1 basal to the clade of all genotype II sequences. There was also a tendency for isolates to cluster according to their country/region of origin.

To analyze the position of Tanzania/Rukwa/2017/1 with respect to the other genotype II viruses at higher resolution, we assembled 29 genotype II genomes, including Tanzania/Rukwa/2017/1, together with an outgroup sequence from genotype I (isolate 74/Ss/2008 from Sardinia, Italy). We then aligned, trimmed the alignment (BMGE with options -t DNA -g 0.0 -h 0.4 -b 10) and inferred a phylogenetic tree with FastTree as above. The resulting cladogram is presented in Fig. 5, annotated with local SH-like support values (ratios) obtained over 1000 re-samplings.

## Discussion

As a result of the recent introduction to Georgia and rapid subsequent dissemination North to the Russian Federation and subsequently East to China and Southeast Asia and West as far as East Germany, genotype II p72 group ASFV represents by far the most geographically widespread of the twenty-four viral genotypes that are currently known^37,38^. Although this virus group is of major concern as the cause of a serious on-going pig pandemic, our study provides the first full genome sequence of a genotype II ASFV from Africa. Our analyses demonstrate close similarity of the Tanzanian sequence to viral sequences from Georgia (2007), Poland (2017) and China (2018), consistent with widespread dissemination of genotype II ASFV following its introduction into Georgia from southeastern Africa in 2007. Based on genotyping and whole genome sequencing, the Georgia 2007/1 virus is believed to have originated in South East Africa, but the exact location within the range of this genotype which includes Mozambique, Malawi, Zambia, southern Tanzania and Madagascar, is not known^8,9^. The divergent sequence of the K177R gene in Rukwa/2017/1 suggests that Tanzania is not the source of the introduction.

The Tanzanian genome determined in this study is relatively closely related to other genotype II viruses that are ultimately derived from Georgia/2007/1, as indicated by the clustering of the Tanzanian genome together with these viruses in a maximum likelihood tree constructed from all publicly available completed ASFV genomes (Fig. 4). and a p72 sequence-based tree (Fig. 5). The one exception to this is a virus isolated from a wild boar in Estonia representing an outlier. In addition to a deletion that removes ORFs located within multicopy gene families located at the 5ʹ end, resulting in reduced virulence, the Estonian virus also contains some unusual rearrangements^36^. The genotype II ASFV from Tanzania exhibits a high level of similarity over the entire length of the genome with the recently re-assembled and annotated Georgia 2007 isolate genome^34^ a Polish genotype from 2017^31^ and a 2018 Chinese isolate^28^. The analysis provides strong evidence that among the set of available full-genome sequences of genotype II, Tanzania/Rukwa/2017/1 is the closest to the phylogenetic divergence point (representing the most likely recent common ancestor) between genotypes I and II. The Tanzania/Rukwa/2017/1 virus was isolated > 1000 km from the southeast African locations thought to have been the likely source of ASFV introduced to Georgia in 2007 through infected pork products (for example, 1270 km from Beira, Mozambique, 1920 km from Maputo, Mozambique, and approximately 2000 km from Antananarivo, Madagascar)^8^. We, therefore, presume that as more ASFV genotype II genome sequences are determined from other parts of the extensive geographical range of this ASFV subtype, even more closely related genomes to Georgia/2007/1 will likely be discovered. Detailed analysis indicates that, although genotype II viruses are highly related to each other, Tanzania/Rukwa/2017/1 shows molecular divergence from Georgia 2007/1, and other group II genomes in the form of indels and a limited number of SNPs. This presumably relates to the fact that mutations differentiating Tanzania/Rukwa/2017/1 from Georgia 2007, are likely to have accumulated over a longer timescale, than the 13 years that have elapsed since the introduction of ASFV to Georgia in 2007.

The pattern in which the majority of the mutations separating Tanzania/Rukwa/2017/I from other genotype II viruses are inserts/deletions (indels) rather than SNPs has been noted previously when all available ASFV genomes were compared^39^. The reason for this mode of genome evolution being more prevalent is not fully understood. However, the virus encodes a proof-reading enzyme 3ʹ to 5ʹ exonuclease, which possibly represents an adaptation to survival in the oxidizing environment of the macrophage, and may contribute to the relative paucity of SNPs (reviewed by^19^). At the same time, the virus also encodes a promiscuous DNA ligase, which may facilitate the generation of indels through non-homologous end joining^19^, although there are some conflicting reports in the literature regarding the fidelity of this enzyme which may be explained by conditions used for in vitro assays.

The organization of the terminal multicopy gene families may also contribute to the high observed frequency of evolution through genomic deletions in ASFV. Loss of certain open reading frames in the multicopy families has been associated with reduction of virulence in domestic pigs, including in recent genotype II isolates from wild boar and domestic pigs^36,40^. Such rearrangements appear to be frequent in genotype II isolates originating from wild boar^36,41^. However, despite the fact there are multiple copy number and potential coding differences in the MGFs (mainly reductions) that differentiate Tanzania/Rukwa/2017/1 from Georgia 2007 and recently modified Eurasian ASF genomes that have re-arranged subsequent to the introduction of this virus to Georgia, the Tanzanian genotype II ASFV that we have sequenced, nonetheless remains sufficiently virulent to cause disease, and was isolated from a clinically reacting domestic pig.

Some genes of known function are entirely conserved throughout the genotype II group of viruses, for example, p30 (CP204L) is 100% conserved, with no SNPs. This gene has been shown to be key to viral replication^42^. The p72 major surface protein gene (B646L) is also 100% conserved between Tanzania/Rukwa/2017/I and the Georgia reference MK645909. This is an important finding given that the primers used for diagnosis (PPA1 and PPA2) and C-terminal sequencing by genotyping (e.g. P72-U and P72-D primers) are designed from this gene that encodes a major viral surface protein. However, by contrast, certain unique ORFs appear to be absent from Tanzania/Rukwa/2017/1, including ASFV_Ch_ACD_00120, which is present in Georgia 2007/1 progeny viruses. This ORF is currently of unknown function, but is presumably not essential for virus survival. The insertion of 16 bases that creates a tandem repeat (aaaaaaataaacaaca) to the p177R locus encoding the K177R structural protein, does not affect the coding sequence of the protein, but might play a role altering expression by modulating transcription or translation.

The annotation of Tanzania/Rukwa/2017/1 revealed 188 putative open reading frames compared to 185 coding sequences (CDS) identified in the annotated Chinese virus genome. However, three of the Tanzania/Rukwa/2017/1 CDS lacked in-frame ATG codons near the N terminal end of the ORF and were therefore annotated as pseudogenes. It is difficult to specify a precise ‘core number’ of coding sequences that are common to all genotype II ASFV viruses, since many of the genomes in the public databases are not yet fully annotated.

Virus isolation in tissue culture remains a gold standard for confirmation of viruses in samples. Given that the virus used in this study is hemadsorbing, a combination of virus isolation and hemadsorption was used as a confirmatory test for the sample and increased the virus titre^43^. The Tanzania/Rukwa/2017/1 virus was passaged in culture only once to minimize the risk of mutations, and sequencing was performed on total DNA extracted from ASFV infected porcine PBMCs. There were no additional procedures used to enrich for viral sequences or reduce the host DNA contamination. This process provided sufficient viral DNA to assemble an almost complete genome, excluding the terminal repeats, as a single contig, using only the medium-throughput Illumina MiSeq platform. In the future, ‘deep sequencing’ of specific loci, using a platform with higher throughput, such as Illumina Hiseq, would provide information on micro-variation that may be present at low levels within the genomes of ASFV field isolates.

Data has recently been published suggesting that p72 genotype II viruses may be actively transmitted from the warthog sylvatic cycle to domestic pigs via *Ornithodoros* ticks at the wildlife domestic pig interface in Mozambique^9^, relatively close to the presumed origin of the genotype II ASFV that was anthropogenically transported to Georgia in 2007. By contrast genotyping using the polymorphic p72 and CVL genes, to type ASFV circulating in Zambia was consistent with one of the two major distinct genotypes representing a recent introduction of genotype II viruses from Tanzania^44^. A comparative analysis including all the public sequences for East-African isolates (including Madagascar and Mauritius) as well as the resequenced Georgia 2007/1 isolate (FR682468.2) and based on the commonly-used genotyping markers p72/B646L, CVR/B604L, p30/CP204L and p54/E183L seen in Supplentary Figures S1–4, revealed three distinct types among East African isolates with p72 genotype II: (1) the Tanzania/Rukwa/2017/1 and Georgia/2007/1 clade that also includes a vast majority of the East African isolates; (2) a group of two ASFV isolates (ZAM/14/Chipata with CVR accession LC174766.1 and ZAM/2017/Chipata/1 with CVR accession LC322009.1) circulating in the Chipata district of Zambia (which differed at all three loci), and the recently described viruses originating from the warthog-tick sylvatic cycle in Mozambique. The tetrameric CVR patterns in Tanzania/Rukwa/2007/1 and in Georgia 2007/1 are identical (BNDBNDBNAL) and correspond to the overwhelming majority of genotype II viruses^9,45^. The Tanzanian Rukwa/2017/1 virus is thus confirmed as being relatively close to the African virus that was the precursor of Georgia/2007/1, with the 16 bp insertion immediately upstream the p22/KP177R gene representing a notable point of difference. In addition, some MGFs differed in length and copy number, and Rukwa/2017/1 was further differentiated from Georgia/2007/1 by a synonymous SNP in the p30/CP204L coding sequence and SNPs in other single-copy loci (see supplementary data for full documentation). This suggests that sequencing of more African p72 genotype II genomes is required to identify with greater accuracy the potential source of the virus that was transported to Georgia.

On an applied note, the observation of molecular divergence between Tanzania/Rukwa/2017/1 and Georgia 2007/1, together with the viruses recently derived from the latter, suggests that live attenuated vaccines derived from Georgia/2007/1 may not always cross-protect against ASFV genotypes derived from different regions of Africa, as it has been observed for phylogenetically distant ASFV strains within the ASFV p72 genotypes^46^.

Understanding the phylogeography and evolutionary dynamics of ASFV genotype II in the endemic areas of Africa encompassing Mozambique, Malawi, Zambia, southern Tanzania and Madagascar, will require future determination of additional complete genomes. To date, the genome sequence data available for both Genotypes I and II is very heavily biased towards viruses from Eurasia, with only two African genotypes available for genotype I, one from South Africa^24^ and the other from Benin^25^ where the sylvatic cycle is not believed to be involved in transmission. The sequence described herein comprises the sole available genome of an African representative of ASFV genotype II. Therefore, little can be concluded regarding the comparative evolution of the genotype I or II ASFV lineages within sub-Saharan Africa. Common to both p72 genotypes I and II are the rearrangements in the terminally located multicopy gene families, resulting in attenuation, have occurred frequently as these viruses adapt to new environments following the transfer out of Africa to Eurasia. This has occurred after adoption of a novel *Ornithodoros* tick vector in the case of genotype I, and infection of wild boar populations in the case of genotype II. A more comprehensive analysis of the comparative evolution of ASFV genotypes I and II, which have assumed global importance due to their transportation to Eurasia and Latin America with resulting major impact on pig production and pig keepers, will become increasingly possible as additional ASFV genomes from the African continent are sequenced and annotated.

## Materials and methods

### Sample processing

**Figure 6:**
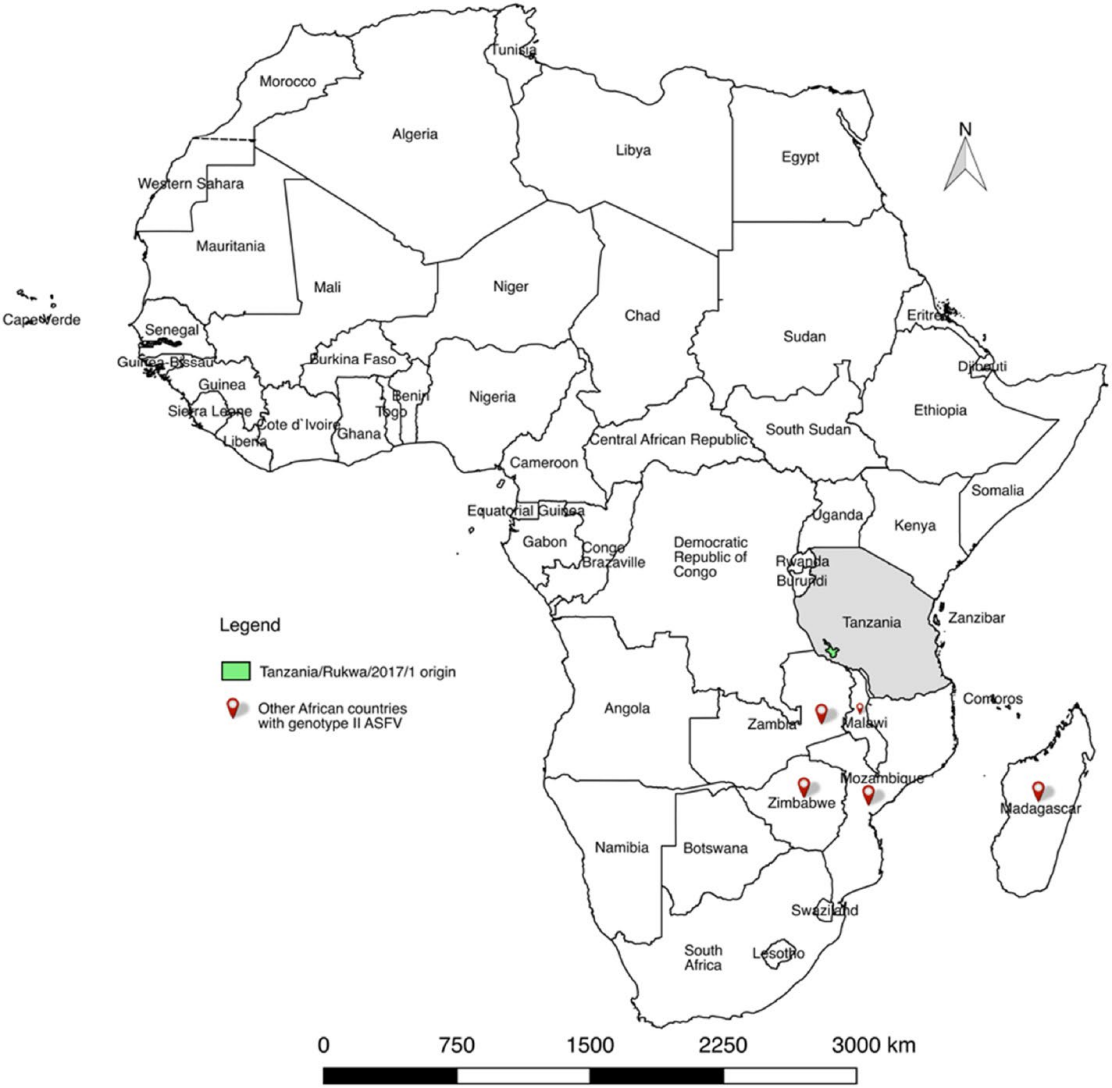
Map of Tanzania showing the location where ASFV samples were collected in this study. The map also shows other locations within South-eastern Africa from where other genotype II ASF viruses were reported. This map was constructed using QGIS software version 2.18.14 freely downloaded from https://qgis.org/en/site/.

Tissue samples used for this study were collected from a year-old female pig that died following a 2017 ASF outbreak in the South-western highland region of Tanzania, in the Sumbawanga rural district of the Rukwa region (Fig. 6). The study maps (Figs. 1 and 6) were generated using QGIS software version 2.18.14 (Las Palmas) using freely available data for maps from DIVA-GIS at http://www.diva-gis.org. The GPS coordinates of the sampling site were 8.85467 S, 31.90858 E. Tissues collected included spleen, heart, lung, mesenteric lymph nodes, liver and kidney samples. Having been demonstrated by PCR to be heavily infected, a spleen sample was used for preparation of a tissue lysate for virus isolation in culture.

### Tissue processing

A sterile surgical blade was used to excise a small fragment of spleen tissue (~ 1 g).

The tissue slice was placed on a sterile sieve positioned on top of a sterile dish prior to being crushed using a pestle allowing the debris to remain on the sieve and the supernatant to pass onto the dish. The sieve was rinsed using 500 µl sterile PBS (pH7.0) containing 5 µg/ml gentamycin sulfate (Bio Whittaker) allowing the lysate to drain onto the dish. The tissue lysate containing PBS was then pipetted out of the dish and transferred into a sterile 1.5 ml Eppendorf tube. The lysate was centrifuged at 14,000 rpm for 10 min to remove tissue debris and the resultant supernatant was pipetted out and transferred into a sterile 1.5 ml Eppendorf tube and stored at − 20 °C. The lysate was used for both extraction of genomic DNA for diagnostic confirmation, for genotyping and as an inoculum for virus isolation.

### Genotyping of the field virus isolate

To determine the p72 genotype, we performed partial sequencing of the C-terminal region of B646L gene that encodes the major capsid protein p72 using primers p72-U (5ʹGGCACAAGTTCGGACATGT3ʹ) and p72-D (5ʹGTACTGTAACGCAGCACAG3ʹ) which amplify a 478 bp region routinely used for initial genotyping of ASFV isolates^21^. The purified PCR amplicon was commercially sequenced using the Sanger method by Macrogen (South Korea).

### ASFV virus isolation

Peripheral blood mononuclear cells (PBMCs) were prepared from fresh porcine blood using standard procedures and ASFV was isolated as described by^47^. Briefly, 0.5 × 10^6^ cells in a 2 ml volume of the complete RPMI were seeded into each well of a 6-well Corning Costar TC-Treated culture plate manufactured by Sigma-Aldrich. The cells were incubated in a humidified chamber at 37 °C and 5% CO_2_ for 3 days to differentiate mononuclear cells into macrophages. They were then inoculated with the thawed tissue suspension (obtained from the tissue processing step above) at a multiplicity of infection (MOI) of 1:10 and incubated for an additional 24 h. 50 µl of 1% autologous red blood cells diluted in sterile PBS (pH 7.0) was added to each well and the plate was examined for evidence of hemadsorption over a 6-day period. The ASFV isolates were blind passaged once.

### DNA extraction

DNA from single passaged porcine PBMC was isolated using the DNeasy Blood and Tissue Kit (Qiagen, Hilden, Germany) following the manufacturer's instructions. Quality and quantity of DNA was assessed using Nanodrop and Qubit spectrophotometry and verified by agarose gel electrophoresis. The total DNA extracted from ASFV infected porcine PBMCs was used to prepare libraries for sequencing without further ASFV DNA enrichment procedure or steps to eliminate DNA host contamination.

### Nextera library preparation using DNA extracted from virally infected PBMC

Library preparation for sequencing was performed using a Nextera XT DNA Library Prep Kit (Illumina, USA) according to the manufacturer’s preparation manual.

### ASFV genome sequencing

An Illumina MiSeq machine was used for the sequencing. Briefly, the genomic library was normalized following the manufacturer’s instructions outlined in the Nextera XT protocol. The flow cell used for the sequencing run was thawed at room temperature together with the sequencing reagents. Thawed reagents were pipetted to mix and 5 μl of the library were transferred into a new tube. The library was diluted to the loading concentration of the sequencing system following the manufacturer’s instructions.

### Genome assembly

The paired reads that were analysed were first trimmed using Trimmomatic (version 0.38) to remove adapter content and low-quality read ends. The exact trimming steps were as follows: ILLUMINACLIP:NexteraPE-PE.fa:2:30:12.LEADING:10.TRAILING:10 SLIDINGWINDOW:4:15 MINLEN:20. Porcine reads were removed by alignment to *Sus scrofa* data in GenBank. This exercise resulted in overall alignment rates of 94.82% for paired reads and 91.44% for unpaired survivors. Virus genome assembly proceeded with the reads that failed to align to the *Sus scrofa* reference using a total of 473,890 paired reads plus 72,928 unpaired reads. We used Unicycler version 0.4.7, which on short-read data acts as a wrapper and enhancer for SPAdes (version 3.13.0). Results with or without the "–linear-seqs 1" option did not differ. We combined all unmapped reads as the input to Unicycler (options '-1', '-2' and '-s' for paired reads 1, paired reads 2 and unpaired reads respectively). The best k-mer size for the assembly was 127, which yielded 505 contigs. The longest assembled segment we obtained was of length 183,186 bp. As part of the Unicycler pipeline, all segments were further polished by Pilon (version 1.23). The final assembly file contained 160 sequences. The resulting longest contig of length 183,186 bp is presented herein as the Tanzania/Rukwa/2017/1 genome.

### Phylogenetic analysis

Whole-genome ASFV sequences from Europe, Africa and Asia were downloaded from the European Nucleotide Archive (ENA) for the purpose of phylogenetic analysis of the Tanzania/Rukwa/2017/1 virus genome. After adding the Tanzania/Rukwa/2017/1 sequence, we generated a multiple sequence alignment using mafft version 7.453 (options -memsave -fft -maxiterate 2 -randomseed 148,345). In order to trim the alignment from its gappy (> 10% gaps) and saturated (entropy h > 0.5) sites, we then used BMGE v.1.12 (options -t DNA -g 0.1 -h 0.5 -b 10), which enabled reduction from 212,659 sites to 169,170 sites suitable for phylogenetic inference. We proceeded using FastTree version 2.1.11 (multi-threaded executable with OpenMP) to infer a phylogenetic tree using the GTR model of nucleotide evolution and a discrete gamma model with 20 rate categories (options -gtr -gamma -spr 4 -mlacc 2 -slownni for more exhaustive NNI steps during maximum-likelihood topology optimization).

### Biosafety

This study was conducted at the Biosciences East and central Africa (BecA) Hub (operating under the umbrella of the International Livestock Research Institution): a regionally approved institution to work with ASFV. All experiments were performed in a biosafety level two laboratory in a biosafety cabinet dedicated for this work.

### Ethics statement

African Swine Fever (ASF) is listed by OIE as a notifiable disease of swine at national, regional and international levels hence no ethical approvals were required for the investigation reported herein. Relevant permits to engage with farmers and collect samples were obtained from regional offices.

## Supplementary Information

Supplementary Information 1.

Supplementary Information 2.

Supplementary Information 3.

Supplementary Information 4.

Supplementary Information 5.

Supplementary Information 6.

## Data Availability

The full genome assembly generated from this study has been deposited in the INSDC databases through the European Nucleotide Archive (ENA) under accession number LR813622. The ENA URL is http://www.ebi.ac.uk/ena/data/view/LR813622. The study ID pertaining to the present publication is PRJEB38524, the sample ID for the sequenced isolate is ERS4590719 and the assembly accession is GCA_9038195.
