# Supplementary figures and images for: The first genotype II African swine fever virus isolated in Africa provides insight into the current Eurasian pandemic
Source: Sci Rep. 2021 Jun 22;11:13081. doi: 10.1038/s41598-021-92593-2 (PMC8219699; doi:10.1038/s41598-021-92593-2)

Tree scale: 0.001

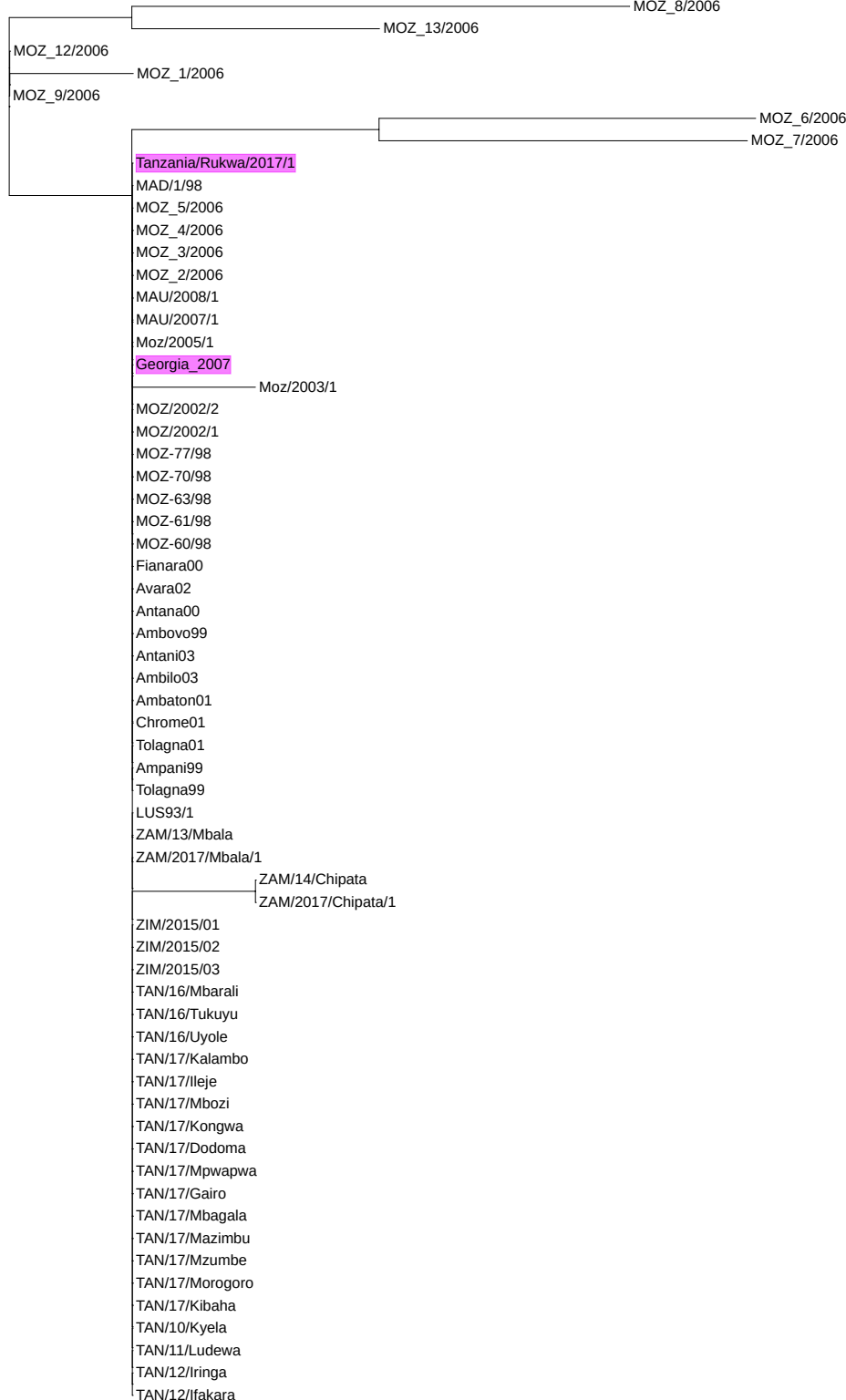

Supplement: Supplementary file 1 — Supplementary Information 1. [file 41598_2021_92593_MOESM1_ESM.pdf]

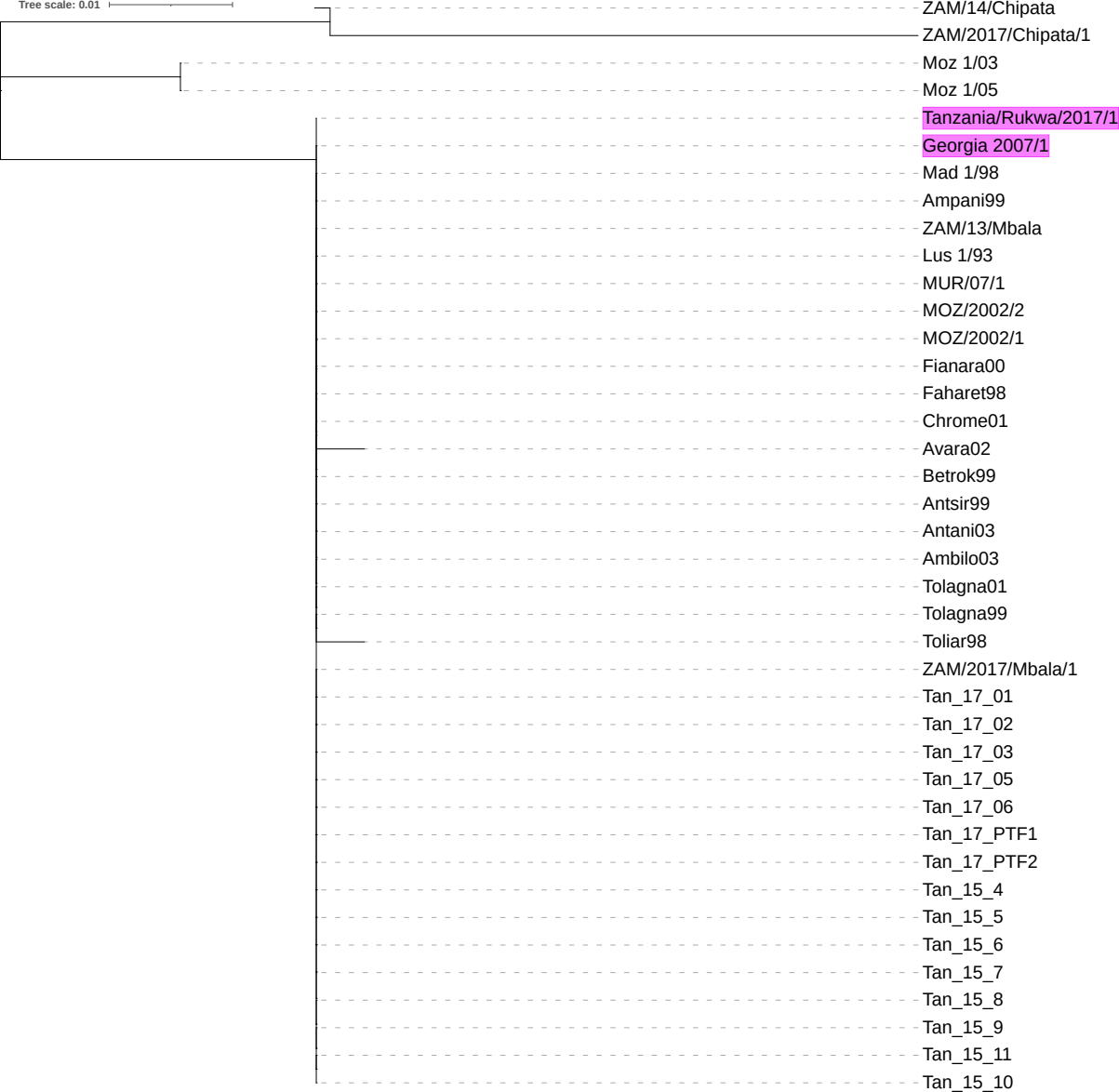

Supplement: Supplementary file 2 — Supplementary Information 2. [file 41598_2021_92593_MOESM2_ESM.pdf]

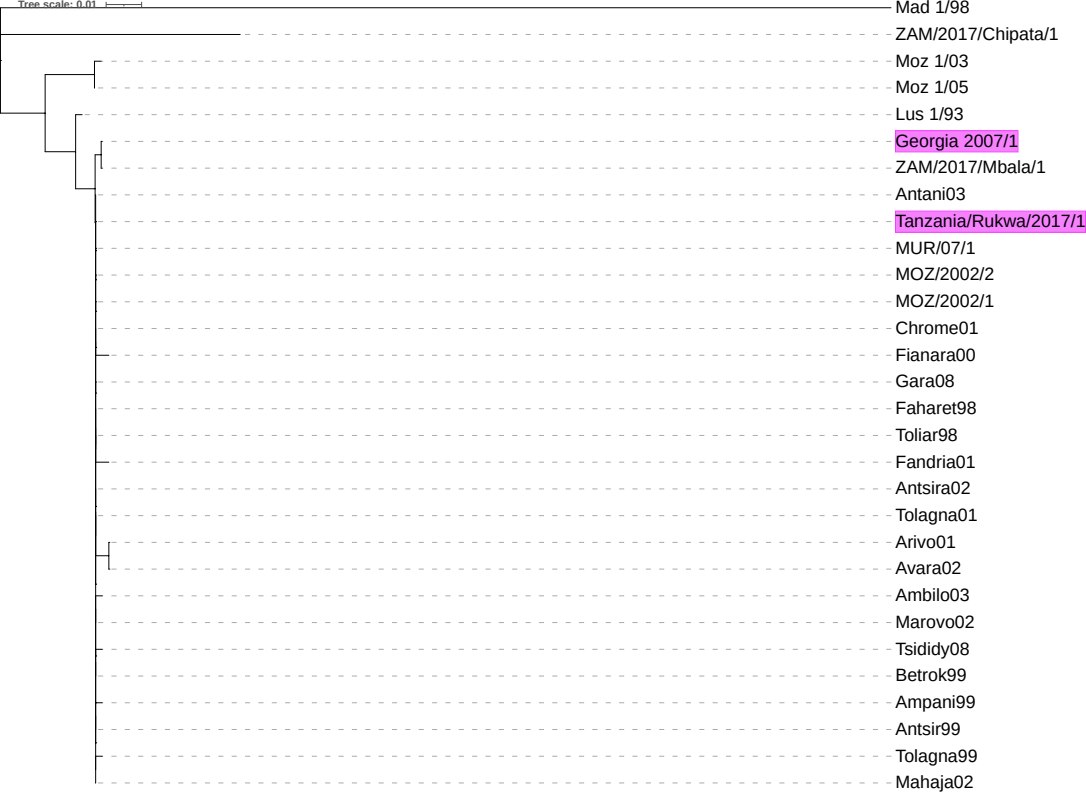

Supplement: Supplementary file 4 — Supplementary Information 4. [file 41598_2021_92593_MOESM4_ESM.pdf]
