# Supplementary material for: The first genotype II African swine fever virus isolated in Africa provides insight into the current Eurasian pandemic
Source: Sci Rep. 2021 Jun 22;11:13081. doi: 10.1038/s41598-021-92593-2 (PMC8219699; doi:10.1038/s41598-021-92593-2)
Supplement: Supplementary file 3 — Supplementary Information 3. [file 41598_2021_92593_MOESM3_ESM.pdf]

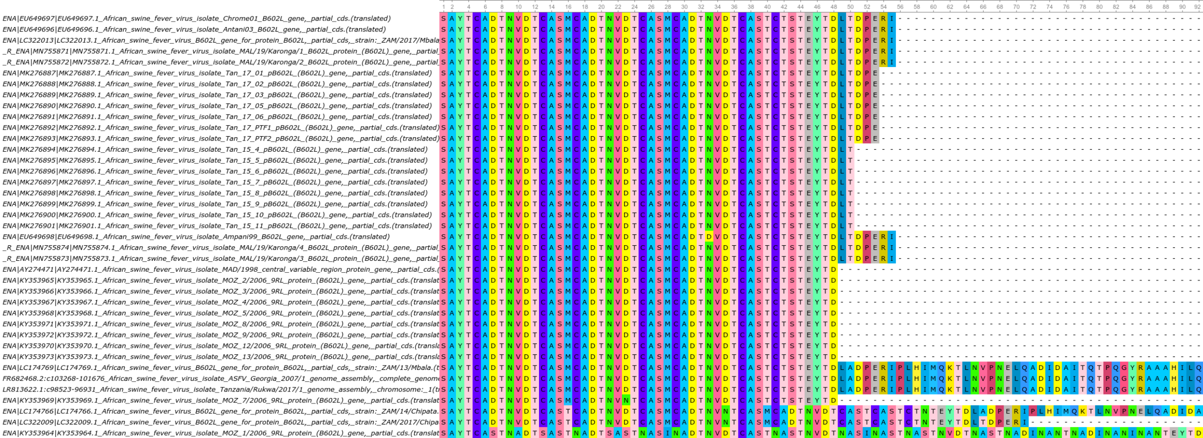

ENA|EU649697|EU649697.1_African_swine_fever_virus_isolate_Chrome01_B602L_gene,_partial_cds.(translated)
ENA|EU649696|EU649696.1_African_swine_fever_virus_isolate_Antani03_B602L_gene,_partial_cds.(translated)
ENA|LC322013|LC322013.1_African_swine_fever_virus_B602L_gene_for_protein_B602L,_partial_cds,_strain:_ZAM/2017/Mbala
_R_ENA|MN755871|MN755871.1_African_swine_fever_virus_isolate_MAL/19/Karonga/1_B602L_protein_(B602L)_gene,_partial
_R_ENA|MN755872|MN755872.1_African_swine_fever_virus_isolate_MAL/19/Karonga/2_B602L_protein_(B602L)_gene,_partial
ENA|MK276887|MK276887.1_African_swine_fever_virus_isolate_Tan_17_01_pB602L_(B602L)_gene,_partial_cds.(translated)
ENA|MK276888|MK276888.1_African_swine_fever_virus_isolate_Tan_17_02_pB602L_(B602L)_gene,_partial_cds.(translated)
ENA|MK276889|MK276889.1_African_swine_fever_virus_isolate_Tan_17_03_pB602L_(B602L)_gene,_partial_cds.(translated)
ENA|MK276890|MK276890.1_African_swine_fever_virus_isolate_Tan_17_05_pB602L_(B602L)_gene,_partial_cds.(translated)
ENA|MK276891|MK276891.1_African_swine_fever_virus_isolate_Tan_17_06_pB602L_(B602L)_gene,_partial_cds.(translated)
ENA|MK276892|MK276892.1_African_swine_fever_virus_isolate_Tan_17_PTF1_pB602L_(B602L)_gene,_partial_cds.(translated)
ENA|MK276893|MK276893.1_African_swine_fever_virus_isolate_Tan_17_PTF2_pB602L_(B602L)_gene,_partial_cds.(translated)
ENA|MK276894|MK276894.1_African_swine_fever_virus_isolate_Tan_15_4_pB602L_(B602L)_gene,_partial_cds.(translated)
ENA|MK276895|MK276895.1_African_swine_fever_virus_isolate_Tan_15_5_pB602L_(B602L)_gene,_partial_cds.(translated)
ENA|MK276896|MK276896.1_African_swine_fever_virus_isolate_Tan_15_6_pB602L_(B602L)_gene,_partial_cds.(translated)
ENA|MK276897|MK276897.1_African_swine_fever_virus_isolate_Tan_15_7_pB602L_(B602L)_gene,_partial_cds.(translated)
ENA|MK276898|MK276898.1_African_swine_fever_virus_isolate_Tan_15_8_pB602L_(B602L)_gene,_partial_cds.(translated)
ENA|MK276899|MK276899.1_African_swine_fever_virus_isolate_Tan_15_9_pB602L_(B602L)_gene,_partial_cds.(translated)
ENA|MK276900|MK276900.1_African_swine_fever_virus_isolate_Tan_15_10_pB602L_(B602L)_gene,_partial_cds.(translated)
ENA|MK276901|MK276901.1_African_swine_fever_virus_isolate_Tan_15_11_pB602L_(B602L)_gene,_partial_cds.(translated)
ENA|EU649698|EU649698.1_African_swine_fever_virus_isolate_Ampani99_B602L_gene,_partial_cds.(translated)
_R_ENA|MN755874|MN755874.1_African_swine_fever_virus_isolate_MAL/19/Karonga/4_B602L_protein_(B602L)_gene,_partial
_R_ENA|MN755873|MN755873.1_African_swine_fever_virus_isolate_MAL/19/Karonga/3_B602L_protein_(B602L)_gene,_partial
ENA|AY274471|AY274471.1_African_swine_fever_virus_isolate_MAD/1998_central_variable_region_protein_gene,_partial_cds.(
ENA|KY353965|KY353965.1_African_swine_fever_virus_isolate_MOZ_2/2006_9RL_protein_(B602L)_gene,_partial_cds.(translat
ENA|KY353966|KY353966.1_African_swine_fever_virus_isolate_MOZ_3/2006_9RL_protein_(B602L)_gene,_partial_cds.(translat
ENA|KY353967|KY353967.1_African_swine_fever_virus_isolate_MOZ_4/2006_9RL_protein_(B602L)_gene,_partial_cds.(translat
ENA|KY353968|KY353968.1_African_swine_fever_virus_isolate_MOZ_5/2006_9RL_protein_(B602L)_gene,_partial_cds.(translat
ENA|KY353971|KY353971.1_African_swine_fever_virus_isolate_MOZ_8/2006_9RL_protein_(B602L)_gene,_partial_cds.(translat
ENA|KY353972|KY353972.1_African_swine_fever_virus_isolate_MOZ_9/2006_9RL_protein_(B602L)_gene,_partial_cds.(translat
ENA|KY353970|KY353970.1_African_swine_fever_virus_isolate_MOZ_12/2006_9RL_protein_(B602L)_gene,_partial_cds.(transla
ENA|KY353973|KY353973.1_African_swine_fever_virus_isolate_MOZ_13/2006_9RL_protein_(B602L)_gene,_partial_cds.(transla
ENA|LC174769|LC174769.1_African_swine_fever_virus_B602L_gene_for_protein_B602L,_partial_cds,_strain:_ZAM/13/Mbala.(t
FR682468.2:c103268-101676_African_swine_fever_virus_isolate_ASFV_Georgia_2007/1_genome_assembly,_complete_genome
LR813622.1:c98523-96931_African_swine_fever_virus_isolate_Tanzania/Rukwa/2017/1_genome_assembly,_chromosome:_1(t
ENA|KY353969|KY353969.1_African_swine_fever_virus_isolate_MOZ_7/2006_9RL_protein_(B602L)_gene,_partial_cds.(translat
ENA|LC174766|LC174766.1_African_swine_fever_virus_B602L_gene_for_protein_B602L,_partial_cds,_strain:_ZAM/14/Chipata.
ENA|LC322009|LC322009.1_African_swine_fever_virus_B602L_gene_for_protein_B602L,_partial_cds,_strain:_ZAM/2017/Chipa
ENA|KY353964|KY353964.1_African_swine_fever_virus_isolate_MOZ_1/2006_9RL_protein_(B602L)_gene,_partial_cds.(translat
