# Supplementary material for: The first genotype II African swine fever virus isolated in Africa provides insight into the current Eurasian pandemic
Source: Sci Rep. 2021 Jun 22;11:13081. doi: 10.1038/s41598-021-92593-2 (PMC8219699; doi:10.1038/s41598-021-92593-2)
Supplement: Supplementary file 5 — Supplementary Information 5. [file 41598_2021_92593_MOESM5_ESM.pdf]

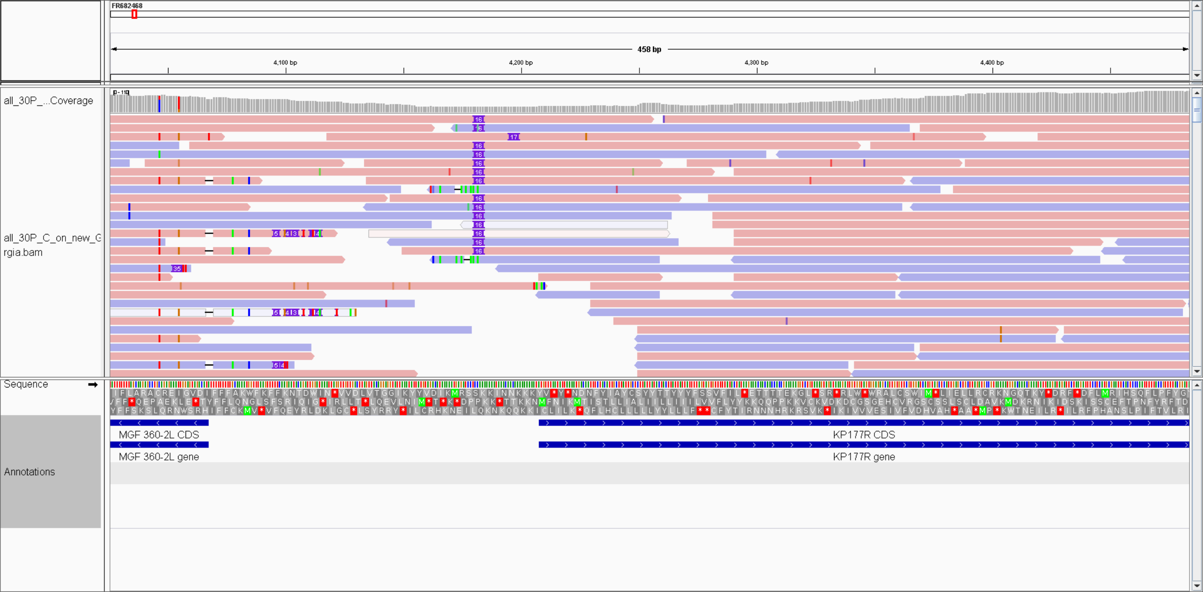
FR682468
458 bp
4,100 bp
4,200 bp
4,300 bp
4,400 bp
all_30P_...Coverage
[0 - 110]
all_30P_C_on_new_G
rgia.bam
Sequence
MGF 360-2L CDS
KP177R CDS
MGF 360-2L gene
KP177R gene
Annotations
